# Loading of Indocyanine Green within Polydopamine-Coated Laponite Nanodisks for Targeted Cancer Photothermal and Photodynamic Therapy

**DOI:** 10.3390/nano8050347

**Published:** 2018-05-19

**Authors:** Fanli Xu, Mengxue Liu, Xin Li, Zhijuan Xiong, Xueyan Cao, Xiangyang Shi, Rui Guo

**Affiliations:** 1 Key Laboratory of Science & Technology of Eco-Textile (Donghua University/Jiangnan University), Ministry of Education, College of Chemistry, Chemical Engineering and Biotechnology, Donghua University, Shanghai 201620, China; xufanli1992@163.com (F.X.); 13262579590@163.com (M.L.); xin_li1991@foxmail.com (X.L.); xiongzj711@sina.com (Z.X.); caoxy_116@dhu.edu.cn (X.C.); 2 State Key Laboratory of Molecular Engineering of Polymers, Fudan University, Shanghai 200433, China

**Keywords:** indocyanine green, laponite, polydopamine, photothermal therapy, photodynamic therapy

## Abstract

The combination of photothermal therapy (PTT) and photodynamic therapy (PDT) in cancer treatment has attracted much attention in recent years. However, developing highly efficient and targeted therapeutic nanoagents for amplifying PTT and PDT treatments remains challenging. In this work, we developed a novel photothermal and photodynamic therapeutic nanoplatform for treatment of cancer cells overexpressing integrin α_v_β_3_ through the coating of polydopamine (PDA) on indocyanine green (ICG)-loaded laponite (LAP) and then further conjugating polyethylene glycol-arginine-glycine-aspartic acid (PEG-RGD) as targeted agents on the surface. The ICG/LAP–PDA–PEG–RGD (ILPR) nanoparticles (NPs) formed could load ICG with a high encapsulation efficiency of 94.1%, improve the photostability of loaded ICG dramatically via the protection of PDA and LAP, and display excellent colloidal stability and biocompatibility due to the PEGylation. Under near-infrared (NIR) laser irradiation, the ILPR NPs could exert enhanced photothermal conversion reproducibly and generate reactive oxygen species (ROS) efficiently. More importantly, in vitro experiments proved that ILPR NPs could specifically target cancer cells overexpressing integrin α_v_β_3_, enhance cellular uptake due to RGD-mediated targeting, and exert improved photothermal and photodynamic killing efficiency against targeted cells under NIR laser irradiation. Therefore, ILPR may be used as effective therapeutic nanoagents with enhanced photothermal conversion performance and ROS generating ability for targeted PTT and PDT treatment of cancer cells with integrin α_v_β_3_ overexpressed.

## 1. Introduction

Photothermal therapy (PTT) and photodynamic therapy (PDT) are emerging physical tumor treatments utilizing near infrared (NIR) light-absorbing agents which lead to thermal ablation of cancer cells or generate highly reactive oxygen species (ROS) via photosensitizer to ablate tumors [1,2]. PTT and PDT possess several advantages, such as minimal invasion, high therapeutic efficacy, limited side-effects, selective localized treatment and reproducible properties [3,4], and hence have received much attention in recent years [5,6]. Until now, a variety of materials has been explored as PTT or PDT agents due to their high absorption in the tissue-transparent NIR wavelength range, including organic fluorescent dyes [7], gold nanorods [8], CuS nanoparticles (NPs) [9], polymer NPs [10], carbon nanomaterials [11], etc. [12,13]. However, fluorescent dyes may be removed rapidly from the systemic circulation and lack specificity to a tumor, and inorganic photothermal agents have potential long-term toxicity due to the difficulty of degrading in the body [14]. Therefore, exploiting biocompatible and targeted therapeutic nanoagents with enhanced photothermal conversion capability and ROS generation ability to amplify PTT and PDT treatments remains challenging.

Indocyanine green (ICG) is a clinical infrared imaging agent approved by the U.S. Food and Drug Administration (FDA), and has been applied in optical imaging of human vasculature, tissue and cells due to its biocompatibility and unique optical properties [15]. Due to strong absorption at 780 nm, ICG can effectively convert absorbed NIR optical energy into heat for PTT [16] and produce ROS for PDT [17] under NIR laser irradiation [18]. Nevertheless, the application of ICG in tumor phototherapy is limited by its tendency to aggregate, rapid degradation in aqueous solution [19], poor photo-stability and non-specific binding to proteins [20]. To overcome those limitations, various nanoparticle delivery systems have been developed to encapsulate ICG [21]. Lvet al. used a mesoporous silica (*m*SiO_2_) matrix to load ICG molecules, and demonstrated that loaded ICG displayed a more enhanced photothermal effect than pure ICG [22]. ICG-loaded mesoporous silica NPs also could not only limit the degradation of ICG, but reach and stay at a tumor for a long period of time due to an enhanced permeability and retention (EPR) effect [23]. Hence, loading of ICG within targeting nanocarriers with high efficiency is shown to be an effective way to promote the application of ICG in PTT and PDT treatment.

Laponite (Na^+^_0.7_[(Si_8_Mg_5.5_Li_0.3_)O_20_(OH)_4_]^−^_0.7_, LAP) is a kind of synthetic nanoclay with potential applications in biomedical fields due to its specific structure, good biocompatibility and biodegradability [24]. In the drug delivery field especially, the layered structure of LAP nanodisks (25 nm in diameter and 0.92 nm in thickness) provides sufficient space to encapsulate drug molecules [25]. In our previous work, the anticancer drug doxorubicin (DOX) was loaded in LAP with a high drug-loading efficiency of 94.3%, and the LAP/DOX nanoparticles formed exhibited more enhanced inhibition effect in cell proliferation than free DOX due to its nanosize [24]. Folic acid modified LAP/DOX nanoparticles could specifically accumulate in cancer cells overexpressing folic acid receptors for targeted therapy [26]. Therefore, loading ICG into LAP nanodisks may improve its stability and prolong its circulation time for phototherapy applications. Polydopamine (PDA) is a eumelanin-liked polymer with excellent biocompatibility, a high photothermal conversion efficiency of 40% and good photothermal stability, which render PDA a potential NIR photothermal agent for PTT therapy [27]. PDA could be easily synthesized in alkaline aqueous media with self-polymerization of dopamine (DA) [27]. The PDA film formed could firmly coat on organic [28] or inorganic [29] solid surfaces, and further bind functional ligands with amine or thiol groups via Michael addition or Schiff base reactions in aqueous solution [30]. For example, polyethylene glycol (PEG) brushes can easily be conjugated on the surface of PDA-capped reduced graphene oxide (rGO) to improve its stability in various solvents [31]. Cheng et al. introduced a targeting PEG-folic acid (PEG-FA) on the surface of PDA, and demonstrated that PEG-FA modified NPs exhibited higher antitumor efficacy against cancer cells with high-expression of folic acid receptors in vivo due to FA-mediated targeting [32]. Also, cyclic peptides containing RGD (Arg-Gly-Asp) motif can be modified on the surface of PDA through a PEG chain [33], which can be used to target cells overexpressing α_v_β_3_ [34]. Taken together, the encapsulation of ICG in LAP nanodisks and then the coating of PDA on the surface may improve the stability of ICG, provide additional photothermal conversion efficacy, and facilitate the targeting modification of a nanoplatform.

In this study, LAP nanodisks were firstly used to load ICG, and then PDA was coated on the surface of ICG/LAP by the self-polymerization of DA, followed by the modification of PEG-RGD as targeted agents on the surface. The ICG/LAP-PDA-PEG-RGD (ILPR) NPs formed were characterized by various techniques, and their photothermal conversion capability and ROS-generation ability were also evaluated. Then, MDA-MB-231 cells (a human breast cancer cell line) overexpressing integrin α_v_β_3_ were used as model cells to evaluate the cytotoxicity and the performance of RGD-mediated targeted delivery of NPs. Finally, the PTT and PDT therapeutic effect of ILPR NPs in vitro under an 808 nm laser irradiation were investigated systematically. To the best of our knowledge, this is the first study on the synthesis of ILPR NPs as effective therapeutic agents for targeted PTT and PDT treatment of cancer cells with overexpressed integrin α_v_β_3_.

## 2. Results and Discussion

### 2.1. Synthesis and Characterization of Indocyanine Green/Laponite–Polydopamine–Polyethylene Glycol–Arg-Gly-Asp (ILPR)Nanoparticles (NPs)

#### 2.1.1. Synthesis and Characterization of ICG/LAP

**Scheme 1:**
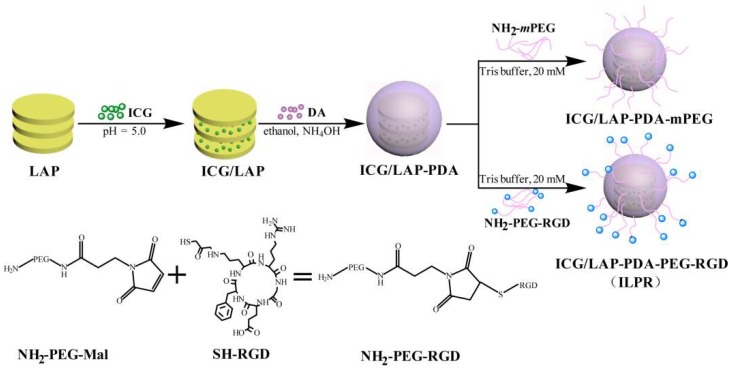
Schematic illustration of the synthesis of ICG/LAP-PDA-*m*PEG and ICG/LAP-PDA-PEG-RGD (ILPR) nanoparticles (NPs).

**Figure 1:**
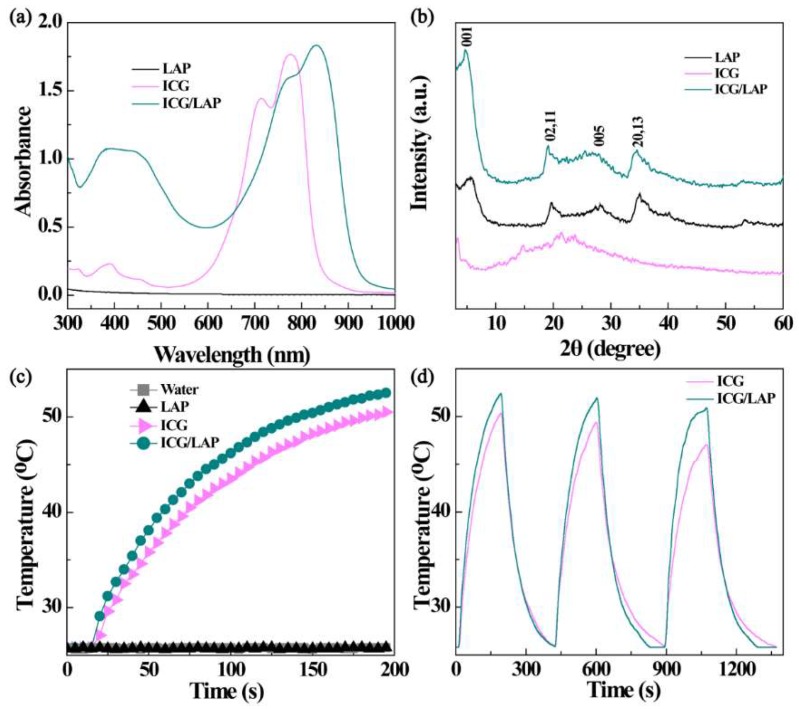
(**a**) Ultraviolet/visible (UV-vis) spectra of LAP, ICG and ICG/LAP; (**b**) X-ray diffraction (XRD) patterns of LAP, ICG and ICG/LAP; (**c**) temperature rising curve of water, LAP, ICG, and ICG/LAP solutions; and (**d**) temperature changes of ICG and ICG/LAP solutions at the same ICG concentration (*C*_ICG_ = 120 μg/mL) under an 808 nm laser irradiation (1.2 W/cm^2^) for 3 cycles (3 min of irradiation for each cycle).

In this study, ICG was firstly loaded in LAP nanodisks in order to increase the stability of ICG in solution, and then PDA were coated on the surface of ICG/LAP to provide additional photothermal conversion efficacy for the nanoplatform and protection for ICG, followed by the modification of RGD–PEG to improve its stability and target cancer cells overexpressing integrin α_v_β_3_ (Scheme 1). Firstly, ICG/LAP NPs were synthesized in acetate solution. Compared with the white color of pure LAP, the centrifugal precipitate of ICG/LAP displayed a dark green color, indicating the encapsulation of ICG in LAP (Figure S1). Ultravoilet/visible (UV-vis) spectroscopy is utilized to further verify the successful loading of ICG in LAP nanodisks (Figure 1a). ICG solution has a characteristic absorption peak at 780 nm, which renders ICG as a NIR light absorber for laser-mediated photothermal therapy [35]. After mixing with ICG, ICG/LAP solution displayed an obvious absorption at around 830 nm in the UV-vis spectra, demonstrating the successful loading of ICG. In comparison with the absorbance of free ICG, the red-shift and broader half-wild of the absorption peak of ICG/LAP is probably attributed to the loading and aggregation of ICG in LAP, which may be more favorable for the photothermal heating with the NIR laser at 808 nm. As shown in Table S1, the mass ratio of ICG to LAP was fixed at 2:9 in following studies with a high ICG loading efficiency of 94.1%. In order to verify the loading mechanism of ICG in LAP, the crystalline structures of LAP and ICG/LAP were investigated by X-ray diffraction (XRD) (Figure 1b), and the diffraction angle and plane spacing data were shown Table 1. After the loading of ICG, only the diffraction angle of (001) plane of LAP shifted from 5.75° to 5.22°, and the corresponding plane spacing increased from 15.37Å to 22.48 Å according to Bragg’s law, which suggest that the incorporation of ICG within LAP is primarily via ion exchange accompanied by intercalation within the LAP interlayer space [36,37]. This result proves that LAP can keep its original crystalline structure after loading with ICG and ICG may mainly intercalate at the (001) plane of LAP. Zeta potential and dynamic light scattering (DLS) measurement illustrated that ICG/LAP NPs kept the size about 120.1 nm with a zeta potential of −16.1 mV (Table 2). Overall, the XRD data suggest that the incorporation of ICG within LAP is primarily via ion exchange accompanied by intercalation within the LAP interlayer space. It is also possible that a portion of ICG can be adsorbed onto the LAP surface via hydrogen bonding or electrostatic interaction, which in turn caused an alteration of the LAP surface potential (from −32.5 ± 3.75 mV to −16.1 ± 1.73 mV, Table 2) after ICG loading.

#### 2.1.2. The Temperature Measurements of ICG/LAP

To evaluate the photothermal efficacy of loaded ICG, the temperature elevation of ICG and ICG/LAP solutions (120 μg/mL) under irradiation of an 808 nm laser (1.2 W/cm^2^, 3 min) were measured (Figure 1c). After laser irradiation, the absolute temperature of aqueous and LAP solutions kept unchanged, while that of ICG and ICG/LAP solutions displayed an apparent increase from 25.8 °C to over 50 °C. And the absolute temperature of ICG and ICG/LAP solutions could be significantly promoted with the increase of ICG concentration (Figure S2), indicating that the temperature enhancement was mainly owing to the strong NIR absorption of ICG. It should be noted that under the same irradiation parameter and ICG concentration, ICG/LAP solution presented a faster and higher absolute temperature increase than free ICG (52.5 °C vs. 50.5 °C, the ambient temperatures = 25.8 °C). This is likely because that after ICG loading, the red-shifted NIR absorbance of ICG/LAP is close to the irradiation wavelength at 808 nm. In addition, the photothermal stability of ICG/LAP was evaluated by 3 cycles of heating and cooling under irradiation by laser at 1.2 W/cm^2^ (3 min for each cycle). As shown in Figure 1d, the highest heated absolute temperature of ICG solution decreased from 50.5 °C to 47.1 °C after 3 cycles of irradiation, indicating the rapid decline of its photothermal heating capability probably because of the serious photo-bleaching of ICG after laser irradiation. In marked contrast, ICG/LAP solution can still keep the highest absolute temperature as 51.0 °C, suggesting its better stability than free ICG under photothermal heating. Moreover, after 3 cycles of irradiation, free ICG solution lost its color and over 60.1% of NIR absorbance, while ICG/LAP solution kept a similar color and its characteristical UV-vis spectrum (Figure S3), demonstrating that ICG/LAP exhibited much better photothermal stability than free ICG. Therefore, the encapsulating of ICG in LAP can enhance its photothermal conversion performance and stability for potential application in photothermal therapy.

#### 2.1.3. Adsorption Isotherms of ICG/LAP

According the reference [38], the adsorption isotherm could be fitted in the Langmuir model wherein the monolayer capacity of the adsorbent can be represented as:

*C*_e_/*C*_s_ = 1/(*C*_max_ × K_L_)+ *C*_e_/*C*_max_

*C_s_* is the adsorbed amount of ICG, *C*_e_ the equilibrium ICG concentration in solution. *C*_max_ the monolayer capacity (208.9 mg·g^−1^) and K_L_ the Langmuir adsorption constant (279.1 L/g). The correlation coefficient of the fitting was *r*^2^_L_=0.9514. It may be noted that a portion of ICG can be adsorbed onto the LAP surface via ion exchange and hydrogen bonding or electrostatic interaction.

#### 2.1.4. Synthesis and Characterization of LAP–Polydopamine (PDA)

**Figure 2:**
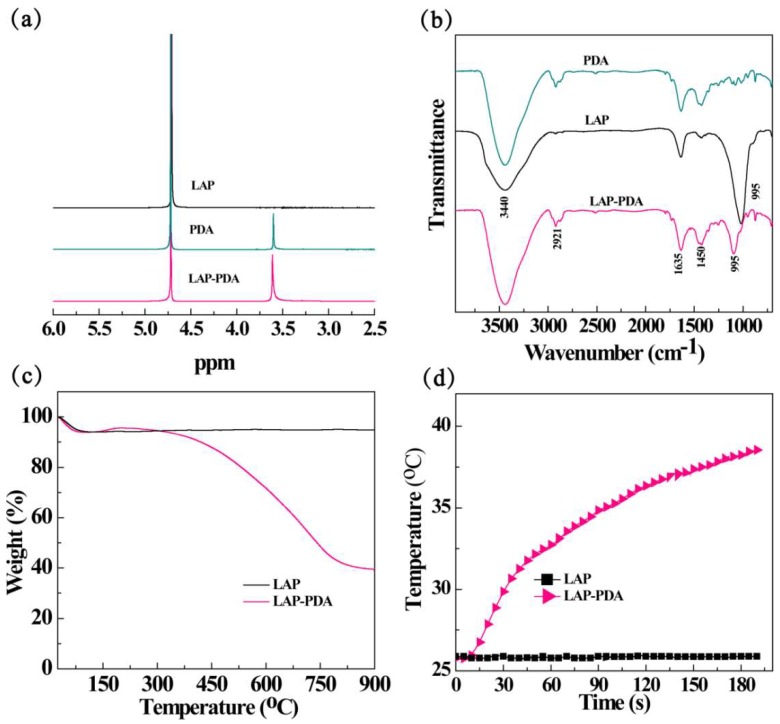
(**a**) ^1^H nuclear magnetic resonance (NMR) spectra in D_2_O and (**b**) Fourier transform-infrared (FT-IR) spectra of LAP, PDA and LAP–PDA; (**c**) TGA curves of LAP and LAP-PDA; (**d**) temperature rising curves of LAP and LAP–PDA solutions (*C*_PDA_ = 300 μg/mL) under an 808 nm laser irradiation (1.2 W/cm^2^, 3 min), respectively.

Dopamine (DA) can spontaneously self-polymerize under alkaline conditions and form adherent PDA film over a wide variety of inorganic or organic surfaces [39]. In this study, DA was mixed with LAP solution for 12 h, and the formed LAP–PDA NPs were collected by centrifugation. The polymerization of DA could be easily speculated by the color change of solution from colorless to dark brown (Figure S4). PDA was confirmed by ^1^H nuclear magnetic resonance (NMR) and Fouriertransform-infrared (FT-IR) spectroscopy. In Figure 2a, the characteristic proton peak related to PDA can be clearly seen at 3.63 ppm, indicating the successful formation of PDA [16]. Figure 2b shows the FT-IR spectra of LAP, PDA and LAP-PDA. After the modification of PDA, the typical peak of Si–O–Si of LAP at 995 cm^−1^ decreased [40], while new bands of PDA appeared in the spectrum of LAP–PDA. The peaks at 2962, 2921 and 2852 cm^−1^ were assigned to the stretching vibrations of C–H, and the peaks at 1635 and 1450 cm^−1^ were induced by aromatic C=N and C=C [41]. Besides, the relatively strong band centered at 3440 cm^−1^ was attributed to the stretching vibrations of –NH and –OH [42]. These results indicated that DA could self-polymerize on the surface of LAP to form LAP–PDA successfully. The formation and relative composition of LAP–PDA was further verified by the thermogravimetric analysis (TGA) (Figure 2c). The weight loss of LAP and LAP–PDA was about 5.9% at 200 °C, which could be ascribed to the removal of adsorbed water. Compared with pristine LAP, LAP–PDA presented a dramatic weight loss of 59.0% in temperature range from 200–900 °C. This should be attributed to the thermal decomposition of PDA, confirming the successful formation of LAP–PDA. According to DLS measurement, LAP–PDA NPs possess a slight bigger size (89.2 nm) than LAP and zeta potential of −32.3 mV (Table S2).

#### 2.1.5. The Temperature Measurements of LAP–PDA

The photothermal conversion ability of LAP–PDA was also investigated by irradiation of 808 nm laser with the power density of 1.2 W/cm^2^ for 3 min (Figure 2d). By contrast with LAP, the temperature of the LAP–PDA solution increased about 12.7 °C compared with ambient temperatures (25.8 °C) after irradiation, demonstrating the photothermal capability of LAP–PDA empowered by coated PDA. In summary, PDA could coat on the surface of LAP nanodisks and endow LAP–PDA with photothermal conversion ability for PTT application and potential surface modification ability.

#### 2.1.6. Synthesis and Characterization of ICG/LAP–PDA

**Figure 3:**
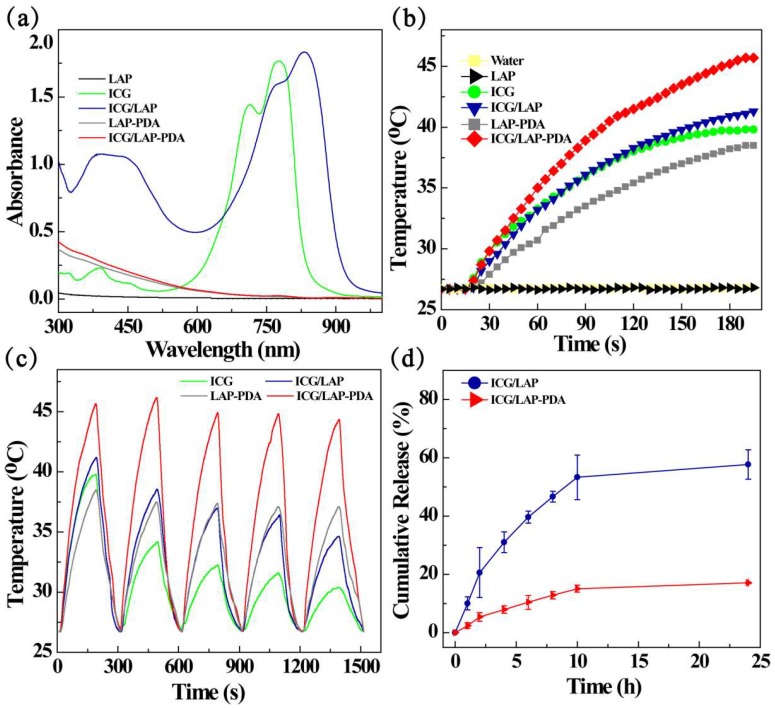
(**a**) UV-vis spectra of ICG, LAP, ICG/LAP, LAP–PDA and ICG/LAP–PDA at the same ICG concentration; (**b**) temperature rising curveof water, LAP, ICG, ICG/LAP, LAP–PDA and ICG/LAP–PDA solutions at the same ICG concentration (*C*_ICG_ = 100 μg/mL) under an 808 nm laser irradiation (1.2 W/cm^2^, 3 min);(**c**) temperature changes of free ICG, ICG/LAP, LAP–PDA and ICG/LAP–PDA solutions at the same ICG concentration (*C*_ICG_ = 100 μg/mL) under irradiation of the 808 nm laser for 5 cycles (1.2 W/cm^2^, 3 min of irradiation for each cycle); (**d**) ICG release from ICG/LAP and ICG/LAP–PDA at 37 °C in the acetate buffers (pH = 5.0).

Then, ICG/LAP–PDA was synthesized by self-polymerization of DA in ICG/LAP solution to improve the photothermal and photodynamic therapeutic capacity via the combination of ICG and PDA. The ICG/LAP–PDA solution formed displayed a typical dark brown color, suggesting the successful synthesis of PDA (Figure S5). The UV-vis spectra of ICG/LAP, LAP–PDA and ICG/LAP–PDA are shown in Figure 3a. By contrast with the appearance of ICG absorption after loading ICG on the surface of PDA in previous studies [4], the strong absorption peak in the NIR region disappeared in the spectrum of ICG/LAP–PDA, suggesting the successful coating of PDA on ICG/LAP. 

#### 2.1.7. The Temperature Measurements of ICG/LAP–PDA

Then, the photothermal conversion ability of ICG/LAP–PDA was evaluated (Figure 3b). After 808 nm laser irradiation for 3 min, the absolute temperature of ICG/LAP and LAP–PDA solutions increased from 25.8 °C to 41.3 °C and 38.5 °C, respectively, whereas the absolute temperature of ICG/LAP–PDA solution with the same concentration of ICG and PDA could be heated from 25.8 upto 45.7 °C, indicating the enhanced photothermal conversion ability of ICG/LAP–PDA in comparison with ICG/LAP and LAP–PDA. The photothermal stability of ICG/LAP–PDA was verified by 5 cycles of heating and cooling under laser irradiation at 1.2 W/cm^2^ (3 min for each cycle) (Figure 3c). After 5 cycles of irradiation, the temperature increment of free ICG solution displayed a dramatic decrease from 14.0 °C to 4.6 °C, while that of ICG/LAP solution declined from 15.5 °C to 8.9 °C, indicating the better stability of ICG/LAP. It is worth noting that almost no decay was found in photothermal performance of ICG/LAP–PDA during 5 cycles of irradiation (19.9–18.9 °C), suggesting that PDA could further improve the photothermal stability of ICG/LAP for photothermal therapy. Moreover, the release curves of ICG from ICG/LAP and ICG/LAP-PDA at pH = 5.0 were studied in Figure 3d. It is clearly shown that under the same condition, ICG/LAP could release 59.0% of ICG, while only about 17.1% of ICG was released from ICG/LAP–PDA. It is speculated that the release of ICG from LAP nanodisks may be stuck by the PDA surface due to both hydrophobic and π–π stacking interactions between ICG and PDA [4]. As a result, the coating of PDA could inhibit the release of ICG and improve the photothermal stability of ICG/LAP–PDA by protecting ICG from photobleaching. Therefore, ICG/LAP-PDA would be an effective therapeutic nanoagent with improved photothermal conversion performance and stability.

#### 2.1.8. Synthesis and Characterization of ILPR

**Figure 4:**
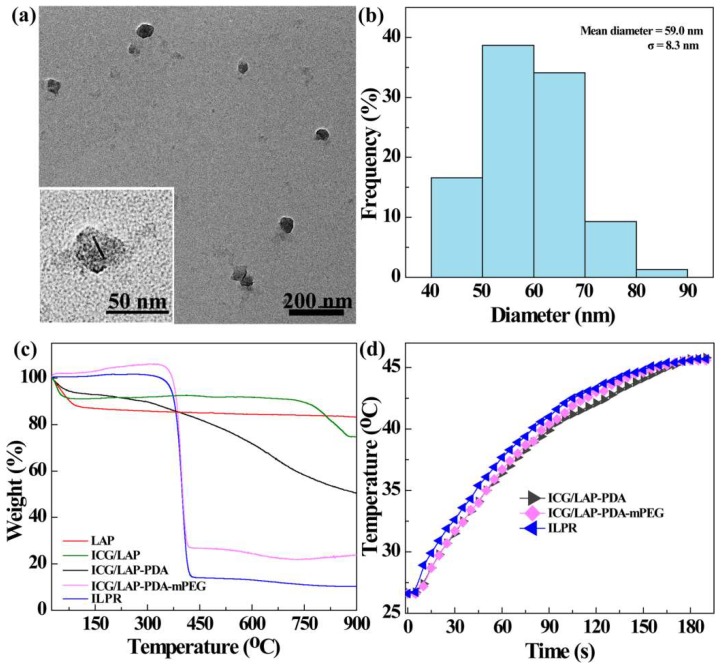
(**a**) The transmission electron microscope (TEM) image and (**b**) corresponding size distribution of ICG/LAP–PDA–*m*PEG; (**c**)the TGA curves of LAP, ICG/LAP, ICG/LAP–PDA, ICG/LAP–PDA–*m*PEG and ILPR, respectively; (**d**) temperature rising curve of ICG/LAP–PDA, ICG/LAP–PDA–*m*PEG and ILPR solutions (*C*_ICG_ = 100 μg/mL) under an 808 nm laser irradiation (1.2 W/cm^2^, 3 min).

To improve the colloidal stability of ICG/LAP–PDA, PEG was modified on the surface of ICG/LAP-PDA through the reaction between amine group of PEG and catechol group of PDA on the surface by the formation of stable Schiff base linkages [33]. The morphology and size distribution of ICG/LAP–PDA–*m*PEG were investigated by transmission electron microscopy (TEM) (Figure 4a,b). The yielded ICG/LAP–PDA–*m*PEG showed uniform spherical shapes, and their average diameter is about 59.0±8.3 nm. From the inserted image, vertical LAP appeared as the black stripe inside of grey PDA NPs, demonstrating the formation of PDA on the surface of ICG/LAP. To endow targeting ability for tumor cells with high-expression of integrin α_v_β_3_, RGD peptides are conjugated on ICG/LAP–PDA using NH_2_-PEG-Mal as linkers. The successful synthesis of NH_2_–PEG–RGD was confirmed by the ^1^H NMR spectrum in Figure S6. The stepwise synthesis of ILPR was confirmed by TGA through comparing the weight loss of intermediate products in the range of 200–900 °C (Figure 4c). By comparing with LAP, ICG/LAP showed a weight loss of 17.3% due to the loading of ICG within nanodisks, which is constituent with the loading capacity calculated by UV-vis results. For ICG/LAP–PDA, there is about 25.1% weight loss by subtracting the residue weight of ICG/LAP at 900 °C, indicating that PDA were coated on the surface of ICG/LAP successfully. After the modification of *m*PEG and RGD–PEG, the weight loss of ICG/LAP–PDA–*m*PEG and ILPR increased to 78.4% and 87.9%, respectively. The TGA result demonstrated that ICG/LAP–PDA–*m*PEG and ILPR were synthesized step by step as scheduled. Figure S7 showed the dispersed state of ICG-LAP-PDA in PBS and ICG/LAP-PDA-mPEG in water, PBS and DMEM, with the modification of *m*PEG, ICG/LAP–PDA–*m*PEG were more stable. Moreover, the hydrodynamic size and surface potential of ICG/LAP–PDA, ICG/LAP–PDA–*m*PEG and ILPR were measured by DLS (Table 2). 

#### 2.1.9. The Temperature Measurements of ILPR

The photothermal conversion abilities of ICG/LAP–PDA–*m*PEG and ILPR solutions were also investigated by irradiation of 808 nm laser for 3 min (Figure 4d). Similar to the ICG/LAP–PDA solution, both ICG/LAP–PDA–*m*PEG and ILPR solution were heated up to about 45.7 °C after irradiation, demonstrating that the modification of *m*PEG and RGD–PEG would not influence its photothermal capability. Finally, PEG–RGD modified ICG/LAP–PDA NPs were successfully synthesized for targeted photothermal and photodynamic therapy. 

To evaluate the function of ICG and PDA in the photothermal and photodynamic treatment of cancer cells, LAP–PDA–PEG–RGD was also synthesized as a control material without loading ICG, and the PDA concentration of LAP–PDA–PEG–RGD was set as the same of ILPR group. The structure of LAP–PDA–PEG–RGD was evaluated by DLS (Table S2) and ^1^H NMR (Figure S8).

### 2.2. Cytotoxicity Assay and Arg-Gly-Asp (RGD)-Targeted Cellular Uptake

**Figure 5:**
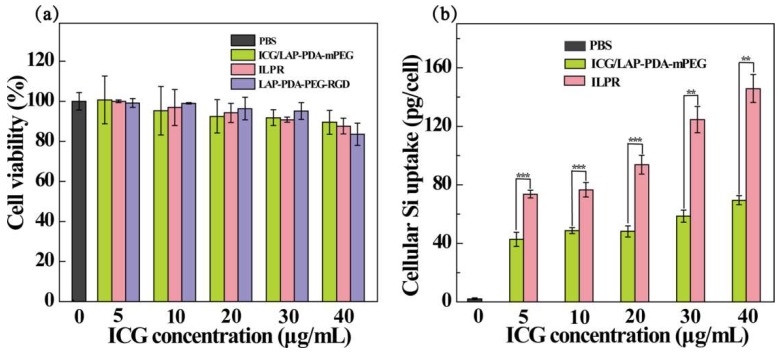
(**a**) CCK-8 viability assay of MDA-MB-231 cells after treatment with LAP–PDA–PEG–RGD, ICG/LAP–PDA–*m*PEG and ILPR NPs at the same ICG concentration (*C*_ICG_ = 5, 10, 20, 30, 40 μg/mL) for 24 h, respectively; (**b**) Cellular uptake of Si of MDA-MB-231 cells after treatment with ICG/LAP–PDA–*m*PEG and ILPR NPs at the same ICG concentration (*C*_ICG_=5, 10, 20, 30, 40 μg/mL) for 6 h, respectively. Phosphate-buffered saline(PBS) buffer was used as control. One-way ANOVA statistical analysis was performed to evaluate the experimental data. A *p* value of 0.05 was selected as the significance level, and the data were indicated with (*) for *p* < 0.05, (**) for *p* < 0.01, and (***) for *p* < 0.001, respectively.

The cytotoxicity of LAP–PDA–PEG–RGD, ICG/LAP–PDA–*m*PEG and ILPR was evaluated by CCK-8 cell viability assay of human breast cancer MDA-MB-231 cells treated with materials at different concentrations (Figure 5a). The cell viabilities of MDA-MB-231 cells treated with materials were above 85% in the studied concentration range from 5 to 40 μg/mL, demonstrating that LAP–PDA–PEG–RGD, ICG/LAP–PDA–*m*PEG and ILPR seem to be non-cytotoxic to cells without laser irradiation. In addition, MDA-MB-231 cells with high-expression of integrin α_v_β_3_ on surface were also used to confirm the RGD-targeted property of ILPR (Figure 5b). It is obvious that MDA-MB-231 cells treated with phosphate-buffered saline (PBS) showed no appreciable Si uptake, while for ICG/LAP–PDA–*m*PEG and ILPR, the celluar Si uptake was apparent and followed a concentration-dependent manner. It is worth noting that the Si uptake of ILPR was significantly higher than that of ICG/LAP–PDA–*m*PEG at the same ICG concentration. The cellular Si uptake of targeted group could be even 2 times higher than that of the untargeted one when the concentration was above 20 μg/mL. These results demonstrated that the modification of RGD enable specific targeting of ILPR to MDA-MB-231 cells with high expression of integrin α_v_β_3_, and afford enhanced cellular uptake due to the RGD-mediated targeting. Therefore, ILPR possesses good cytocompatibility and targeting ability for cancer cells overexpressing integrin α_v_β_3_.

### 2.3. Reactive Oxygen Species (ROS)Production

**Figure 6:**
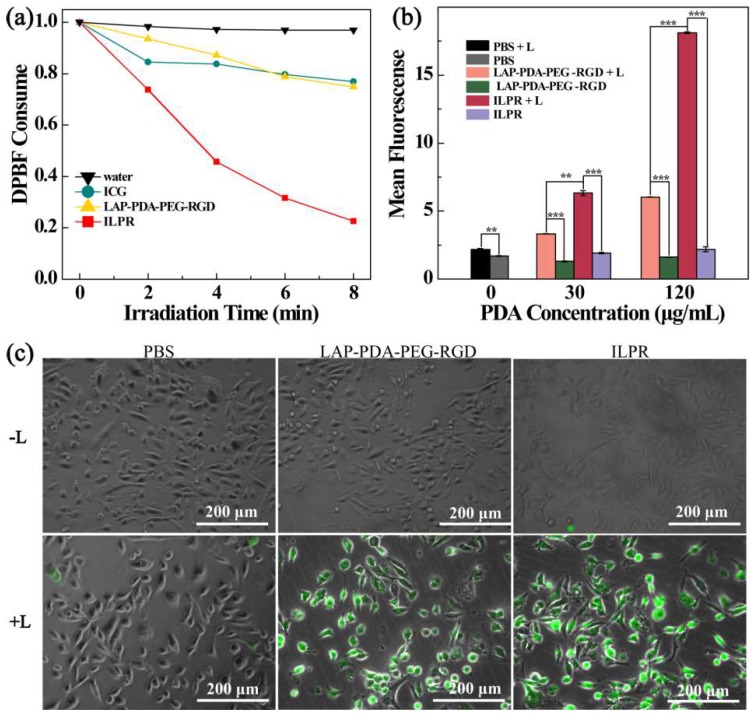
(**a**) Consumption of DPBF (1,3-diphenylisobenzofuran)over time due to ^1^O_2_ generation for water, LAP, ICG, LAP–PDA–PEG–RGD and ILPR aqueous solution with an 808 nm laser irradiation (1.2 W/cm^2^); (**b**) mean fluorescence of DCF in MDA-MB-231cells stained by DCF-H after incubated with LAP–PDA–PEG–RGD and ILPR NPs at the same ICG concentration (*C*_ICG_ = 10, 40 μg/mL) with/without laser irradiation (1.2 W/cm^2^, 5 min), a *p* value of 0.05 was selected as the significance level, and the data were indicated with (*) for *p* < 0.05, (**) for *p* < 0.01, and (***) for *p* < 0.001, respectively; (**c**) fluorescence microscopic images of MDA-MB-231cells stained by DCF-H after the cells incubated with LAP–PDA–PEG–RGD and ILPR NPs (*C*_ICG_ = 40 μg/mL) with/without laser irradiation (+L/-L) (1.2 W/cm^2^, 5 min).PBS buffer was used as control.

Since ROS generation is the key component for PDT, the capability of ILPR in producing ROS was evaluated by utilizing DPBF (1, 3-diphenylisobenzofuran) as a probe to detect the production of cytotoxic ROS under laser irradiation via UV-vis spectroscopy. DPBF can react with the generated ROS, and the absorption intensity of DPBF at 417 nm may decrease with the increase of ROS concentration. As shown in Figure 6a, there is no noticeable change of DPBF absorption in water and LAP under laser irradiation, while the absorption intensity of DPBF in ICG, LAP–PDA–PEG–RGD and ILPR groups decreased gradually, demonstrating their ability to continuously generate ROS overtime under laser irradiation. It should be noted the decrease of DPBF intensity by ILPR (77.4%) is significantly higher than that of ICG (23.1%) and LAP–PDA–PEG–RGD (25.1%), which should be attributed to the combined effect of ICG and PDA. Interestingly, ILPR displayed a continuous high rate of decay of DPBF in comparison with ICG with a decreased rate, probably due to the protection of ICG from photobleaching by LAP and PDA in ILPR. To evaluate the formation of ROS induced in cells under laser irradiation, non-fluorescent dichloro-dihydro-fluorescein (DCF-H) is used as a fluorogenic marker for ROS due to the oxidization of DCF-H to fluorescent DCF by the generated ROS. Figure 6b displays the mean fluorescence of MDA-MB-231 cells treated with ILPR and LAP–PDA–PEG–RGD with/without laser irradiation. It is obvious that for the PBS group with/without irradiation and the materials group without irradiation, the fluorescence intensity of cells is lower than 3.0, suggesting that ROS may not be produced by laser irradiation or materials alone. In contrast, the mean fluorescence of cells treated with ILPR and LAP–PDA–PEG–RGD presented apparent enhancement after irradiation, indicating the laser irradiation-triggered generation of ROS in MDA-MB-231 cells. More importantly, the ILPR group displayed a significant higher mean fluorescence than LAP–PDA–PEG–RGD (*p* < 0.01 at 10 μg/mL, *p* < 0.001 at 40 μg/mL), and the difference between them was enhanced with the increase of ICG concentration, suggesting that the loaded ICG may improve their ability to produce ROS dramatically. In addition, the DCF fluorescence in MDA-MB-231 cells is directly observed by fluorescence microscopy to confirm the production of ROS (Figure 6c). There is no obvious green fluorescence in cells without laser irradiation, verifying that neither of the materials could induce ROS without laser irradiation. Irradiated by an 808 nm laser, MDA-MB-231 cells treated with ILPR and LAP–PDA–PEG–RGD could exhibit strong green fluorescence, demonstrating that ROS can be produced by both materials in live cells with NIR irradiation. Consistent with the flow cytometric analysis, the florescence intensity of cells treated with ILPR is much higher than that of LAP–PDA–PEG–RGD, indicating its enhanced ability to generate ROS. Overall, ILPR may be an effective PDT agent to generate ROS for the photodynamic treatment of cancer cells.

### 2.4. Combination of Photothermal Therapy (PTT) and Photodynamic Therapy (PDT) of Cancer CellsIn Vitro

**Figure 7:**
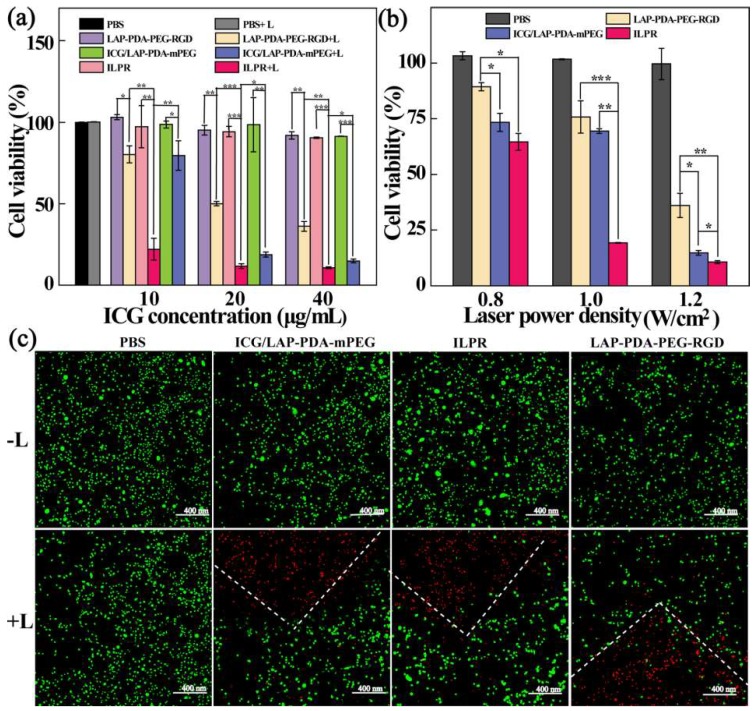
Cell viabilities of MDA-MB-231 cells after incubated with LAP–PDA–PEG–RGD, ICG/LAP–PDA–*m*PEG and ILPR NPs (**a**) at different ICG concentrations (*C*_ICG_ = 10, 20, 40 μg/mL) with/without irradiation of an 808 nm laser (2.5 cm^2^, 1.2 W/cm^2^, 5 min); and (**b**) at the same ICG concentration (*C*_ICG_ = 40 μg/mL) with an 808 nm laser irradiation (2.5 cm^2^, 5 min) at different power densities (0.8, 1.0, 1.2 W/cm^2^), a*p* value of 0.05 was selected as the significance level, and the data were indicated with (*) for *p* < 0.05, (**) for *p* < 0.01, and (***) for *p* < 0.001, respectively.; (**c**) fluorescence microscopic images of Calcein AM and PI co-staining MDA-MB-231 cells after treatment with PBS, LAP–PDA–PEG–RGD, ICG/LAP–PDA–*m*PEG and ILPR at the same ICG concentration (*C*_ICG_ = 40 μg/mL) with/without (−L/+L) irradiation of an 808 nm laser (0.25 cm^2^, 1.2 W/cm^2^, 5 min). PBS buffer was used as control.

To investigate the in vitro photothermal and photodynamic therapeutic effect, the viability of MDA-MB-231 cells incubated LAP–PDA–PEG–RGD, ICG/LAP–PDA–*m*PEG and ILPR NPs with irradiation of an 808 nm laser (2.5 cm^2^) at various ICG concentrations (Figure 7a)and different power densities (Figure 7b) were measured by CCK-8 assay. For the PBS group, the viability of cells with laser irradiation was similar to those without, indicating that the laser irradiation may not hurt cells. When the power density of laser was set at 1.2 W/cm^2^ (Figure 7a), the viabilities of cells incubated with materials at the studied concentration range were higher than 90.0% without irradiation, while the cell viabilities decreased dramatically under irradiation, suggesting the cytotoxicity was produced under NIR light. When the ICG concentration is as low as 10 μg/mL, the viability of cells treated with ILPR decreased to 22.1% under irradiation, which is significantly lower than that of LAP–PDA–PEG–RGD (80.2%, *p* < 0.01) and that of ICG/LAP–PDA–*m*PEG (79.6%, *p* < 0.01). With the increase of material concentration, their inhibition effect increased gradually. The half-maximal inhibitory concentration (*IC*_50_) of ILPR (7.27 μg/mL) was found to be 1.9 times lower than that of ICG/LAP–PDA–*m*PEG (13.7 μg/mL) and 3.1 times lower than that of LAP–PDA–PEG–RGD (22.6 μg/mL), suggesting that the RGD-mediated targeting could enhance their PTT and PDT efficacy to cancer cells with high expression of integrin α_v_β_3_. This result demonstrated that the photothermal and photodynamic therapeutic effect of ILPR is far superior to that of LAP–PDA–PEG–RGD and ICG/LAP–PDA–*m*PEG. When the concentration of ICG was set at 40 μg/mL, the cell viabilities decreased dramatically under irradiation with the increase of laser power density (Figure7b). LAP–PDA–PEG–RGD group displayed the least therapeutic effect among three material groups, due to the lack of ICG as NIR absorbent. At the same laser power density of 1.0 W/cm^2^, the viability of cells treated with ILPR (19.3%) is significantly lower than that of ICG/LAP–PDA–*m*PEG (69.5%, *p* < 0.01), which may be attributed to the RGD-mediated specific targeting of ILPR to MDA-MB-231 cells overexpressing integrin α_v_β_3_. In order to reach a similar inhibition effect as ILPR, either an increased laser power density or a higher concentration of materials is needed for ICG/LAP–PDA–*m*PEG. Therefore, ILPR could specifically target cancer cells overexpressing integrin α_v_β_3_ and effectively exert enhanced photothermal and photodynamic therapeutic efficacy under NIR laser irradiation.

Finally, the therapeutic effect of ILPR was confirmed directly by the fluorescence microscopy observation of MDA-MB-231 cells after staining with live/dead assay kit (Figure 7c). The viability of cells could be easily distinguished by the green fluorescence for live cells and the red fluorescence for dead ones. Without laser irradiation, all cells showed green fluorescence, suggesting the good biocompatibility of LAP–PDA–PEG–RGD, ICG/LAP–PDA–*m*PEG, and ILPR. After 5 min of laser irradiation, the cells treated with PBS still exhibited green fluorescence, indicating that laser alone may not kill cells, which is consistent with the CCK-8 result. For material groups, large amount of cells displayed red fluorescence signal in the laser spot area with clear boundary, while the cells out of that area kept sparkling green fluorescence. This result further demonstrated that ILPR could induce the death of cells by photothermal and photodynamic effects with specific laser irradiation. Taken together, ILPR may become an effective and targeted therapeutic nanoagent for photothermal and photodynamic cancer treatments.

## 3. Experimental Section

### 3.1. Materials

Laponite (Na^+^_0.7_[(Si_8_Mg_5.5_Li_0.3_)O_20_(OH)_4_]^−^_0.7_, LAP) was purchased from Guangzhou Bo Feng Chemical Technology Co., Ltd. (Guangzhou, China). Indocyanine Green (C_43_H_47_N_2_NaO_6_S_2_ > 90%, ICG), dopamine (C_8_H_11_NO_2_·HCl > 98%, DA), *N*, *N*-dimethylformamide (C_3_H_7_NO > 97%, DMF) and 1,3-Diphenylisobenzofuran (C_20_H_14_O > 97%, DPBF) were purchased from J&K Chemical Ltd. (Shanghai, China). *m*PEG-NH_2_ and NH_2_-PEG-Mal (*M*w = 5000) were obtained from Shanghai Yarebio. Co., Ltd. (Shanghai, China). Thiolated cyclic RGD peptide (*M*w = 690.93) was purchased from GenicBio (Shanghai, China). Cell Counting Kit-8 (CCK-8) was obtained from Beyotime Biotechnology (Shanghai, China). Reactive oxygen species assay kit mainly containing dichloro-dihydro-fluorescein diacetate (DCFH-DA) was purchased from Shanghai YEASEN Biotechnology Co., Ltd. (Shanghai, China). All chemicals were used without further purification. MDA-MB-231 cells (a human breast cancer cell line) were acquired from Chinese Academy of Sciences cell bank (Shanghai, China). Dulbecco’s modified eagle medium (DMEM), penicillin, streptomycin, and fetal bovine serum (FBS) were purchased from Hangzhou Jinuo Biomedical Technology (Hangzhou, China). Water used in all experiments was purified using a Milli-Q Plus. 185 water purification system (Millipore, Bedford, MA, USA) with a resistivity higher than 18.2 MΩ·cm. Microsep with a molecular weight cut-off of 10,000 was purchased from Merck Millipore Ltd., (Kenilworth, NJ, USA). Two type of 808 nm laser (FC-808-10W-MM with a beam spot size of 2.5 cm^2^ and DL-808-2000-T with a beam spot size of 0.25 cm^2^) were purchased from Shanghai Xilong Optoelectronics Technology Co. Ltd., (Shanghai, China).

### 3.2. Synthesis of ICG/LAP

LAP (18 mg) was dispersed in 9 mL of acetate buffer (pH = 5.0). Then ICG solution (2 mg/mL, 2 mL) was added into the LAP solution under magnetic stirring for 4 h. An optimized mass ratio of LAP to ICG at 9:2 was employed in this study. The formed ICG/LAP NPs were collected by centrifugation (6000 rpm, 5 min) and rinsing with water 3 times.

### 3.3. Synthesis of the LAP–PDA and ICG/LAP–PDA NPs

We added 11.5 mL of ethanol (98%) and 0.9 mL of ammonia aqueous solution (25–28%) into LAP solution (0.72 mg/mL, 25 mL) under mild stirring at room temperature for 30 min to keep the ultimate volume fraction of ethanol at about 30% [43]. Then 2.88 mL of DA solution (50 mg/mL) was added into the aforementioned mixture under magnetic stirring for 12 h. The LAP–PDA NPs formed were collected by centrifugation in Microsep (Millipore, Billerica, MA, USA) with a molecular weight cut off (MWCO) of 10,000 (8500 rpm, 30 min) and rinsing with water 3 times.

ICG/LAP–PDA NPs were synthesized by self-polymerization of DA in ICG/LAP solution by the similar experimental protocol.

### 3.4. Synthesis of the ILPR NPs

RGD solution (2.4 mg/mL, 1 mL) was dropwise added into NH_2_-PEG-Mal solution (12 mg/mL, 5 mL) under magnetic stirring. The reaction continued for 3 days, and then the reaction mixture solution was dialysed against water using a dialysis membrane with a MWCO of 1000. Then the NH_2_-PEG-RGD solution was added into ICG/LAP–PDA solution (10 mg/mL, 1 mL) in tris buffer (pH = 9.00, 20 mM) under magnetic stirring for 24 h. The formed ILPR was purified by centrifugation in Microsep (Millipore, Billerica, MA, USA) with a MWCO of 10,000 (8500 rpm, 30 min)and rising with water for 3 times to remove excess NH_2_-PEG-RGD, and were lyophilized to preserve in dark for following use. According to above experimental protocol, LAP–PDA–PEG–RGD was synthesized by the modification of NH_2_-PEG-RGD on LAP–PDA NPs and ICG/LAP–PDA–*m*PEG was obtained by the modification of *m*PEG-NH_2_ on ICG/LAP–PDA.

## 4. Conclusions

In summary, we developed a targeted therapeutic agent for photothermal and photodynamic treatment of cancer cells overexpressing integrin α_v_β_3_. In our approach, the ILPR nanoparticles were synthesized by the loading of ICG in LAP nanodisks and then coating these with PDA, followed by the modification of PEG–RGD as targeted agents on the surface. ICG could be effectively encapsulated in LAP with an efficiency of 94.1%, and its photothermal and photostability could be improved dramatically in NPs by the protection of LAP and PDA from photobleaching. The ILPR NPs formed possessed excellent colloidal stability, cytocompatibility, continuously efficient production of ROS in live cells under laser irradiation, and enhanced photothermal conversion ability due to the combined effect of ICG and PDA. More importantly, the ILPR NPs could specifically target cancer cells overexpressing integrin α_v_β_3_, enhance cellular uptake due to RGD-mediated targeting, and exert improved photothermal and photodynamic therapeutic effect to targeted cells upon NIR laser irradiation. Therefore, ILPR NPs may be used as effective therapeutic agents with enhanced photothermal conversion performance and ROS-generating ability for targeted PTT and PDT treatment of cancer cells with overexpressed integrin α_v_β_3_.

## Figures and Tables

**Table 1:** Diffraction angle and plane spacing data of LAP and ICG/LAP from XRD analysis.

Diffraction Plane (hkl)	2θ Peak Position (°)		Plane Spacing (*d*, Å)	
	LAP	ICG/LAP	LAP	ICG/LAP
(001)	5.75	5.22	15.37	22.48
(02, 11)	19.66	19.48	4.51	6.75
(005)	28.22	27.22	3.16	3.27
(20, 13)	34.56	34.44	2.59	2.60

**Table 2:** Zeta potential and hydrodynamic size of LAP, ICG/LAP, ICG/LAP-PDA, ICG/LAP-PDA-*m*PEG and ILPR NPs, respectively.

Materials	Zeta Potential (mV)	Hydrodynamic Size (nm)	Polydispersity Index (PDI)
LAP	−32.5 ± 3.75	62.8 ± 2.74	0.205 ± 0.038
ICG/LAP	−16.1 ± 1.73	120.1 ± 7.52	0.316 ± 0.072
ICG/LAP-PDA	−20.7 ± 1.25	133.0 ± 9.97	0.239 ± 0.044
ICG/LAP-PDA-*m*PEG	−9.14 ± 1.18	136.0 ± 13.6	0.360 ± 0.062
ILPR	−7.33 ± 0.82	147.9 ± 9.97	0.404 ± 0.017

## Supplementary Materials

The following are available online at http://www.mdpi.com/2079-4991/8/5/347/s1.
