# Supplementary material for: Loading of Indocyanine Green within Polydopamine-Coated Laponite Nanodisks for Targeted Cancer Photothermal and Photodynamic Therapy
Source: Nanomaterials (Basel). 2018 May 19;8(5):347. doi: 10.3390/nano8050347 (PMC5977361; doi:10.3390/nano8050347)
Supplement: Supplementary file 1 [file nanomaterials-08-00347-s001.pdf]

Supporting Information

Loading of Indocyanine Green within Polydopamine-Coated Laponite Nanodisks for Targeted Cancer Photothermal and Photodynamic Therapy

FanliXu[1], MengxueLiu[1], XinLi[1], ZhijuanXiong[1], XueyanCao[1], XiangyangShi[1,*] and RuiGuo[1,2,*]

[1] Key Laboratory of Science & Technology of Eco-Textile (Donghua University/Jiangnan University), Ministry of Education, College of Chemistry, Chemical Engineering and Biotechnology, Donghua University, Shanghai 201620, China; xufanli1992@163.com (F.X.); 13262579590@163.com (M.L.); xin_li1991@foxmail.com (X.L.); xiongzj711@sina.com (Z.X.); caoxy_116@dhu.edu.cn (X.C.)

[2] State Key Laboratory of Molecular Engineering of Polymers, Fudan University, Shanghai 200433, China

* Correspondence: xshi@dhu.edu.cn (X.S.); ruiguo@dhu.edu.cn (R.G.); Tel.: +86-21-6779-2750 (R.G.); Fax: +86-21-6779-2306 (ext. 804, R.G.)

Part of experimental section

Characterization Techniques

UV-Vis spectroscopy was obtained using a Lambda 25 UV-Vis spectrophotometer (Perkin-Elmer, USA). The crystalline structures of LAP, ICG and ICG/LAP were characterized by a Rigaku D/max-2550 PC X-ray diffraction (XRD) system (Rigaku Co., Tokyo, Japan) using Cu Ka radiation with a wavelength of 1.54 Å at 40 kV and 200 mA. The scan was performed from 5° to 60° (2θ). The plane spacing of a different diffraction plane (*dhkl*) can be calculated from Bragg's law. ^{1}H NMR spectra were measured on a Bruker AV400 NMR spectrometer. Samples were dissolved in D_2O before measurement. Zeta potential and dynamic light scattering (DLS) measurements were carried out using a Malvern Zetasizer Nano ZS model ZEN3600 (Worcestershire, U.K.) equipped with a standard 633 nm laser. Thermogravimetric analysis (TGA) was applied to characterize the modification of PDA and PEG-RGD on LAP by using a TGA 209 F1 (NETZSCH Instruments Co, Ltd., Selb/Bavaria, Germany). The samples were heated from 25 °C to 900 °C at a rate of 10 °C/min under nitrogen atmosphere. Transmission electron microscopy (TEM) was carried out with a JEOL 2010 analytical electron microscope (Tokyo, Japan) operating at 200 kV. Before performing the measurements, the samples were prepared by putting a drop of diluted NP suspension (6 μL) onto a carbon-coated copper grid and dried in air. For each sample, at least 200 particles in different TEM images were randomly selected and measured and use ImageJ software to calculate the size distribution of NPs. The Si concentrations of samples were analysed by using a Leeman Prodigy ICP-OES system (Hudson, NH). The photothermal property and stability of the NPs were tested according to the literature [39]. For photothermal conversion capacity test, ICG, LAP, ICG/LAP, LAP-PDA, ICG/LAP-PDA, ICG/LAP-PDA-*m*PEG, and ICG/LAP-PDA-PEG-RGD (ILPR) solutions were irradiated using an 808nm laser (0.25 cm^2, 1.2 W/cm^2, 3 min) (Shanghai Xilong

Optoelectronics Technology Co. Ltd., Shanghai, China). The temperature was recorded every 5 s by an online DT-8891 Ethermocouple thermometer (Shenzhen Everbest Machinery Industry Co., Ltd., Shenzhen, China). In order to investigate the photothermal stability, five cycles of repeated laser irradiation were applied to ICG, LAP, ICG/LAP, LAP-PDA and ICG/LAP-PDA solutions. For each cycle, the solution was heated by an 808 nm laser irradiation (0.25 cm^2, 1.2 W/cm^2, 3 min) and followed by a cooling period to room temperature. The solution temperature was recorded every 5 s by thermometer.

Cell Culture and Cytotoxicity Assay

Human breast cancer MDA-MB-231 cells overexpressing integrin $\alpha_v\beta_3$ were cultured and passaged in 25-cm^2 plates with DMEM supplemented with 10% FBS and 1% penicillin/streptomycin under 37 °C and 5% CO_2.

CCK-8 assay was used to quantify the viability of MDA-MB-231 cells after treated with LAP-PDA-PEG-RGD, ICG/LAP-PDA-*m*PEG or ILPR NPs at different ICG concentrations. Briefly, MDA-MB-231 cells were seeded in 96-well plates at a density of 1×10^4 cells/well with 100 μL of fresh DMEM at the day before the experiment. After 24 h, the medium was replaced by 100 μL fresh medium containing PBS (control), LAP-PDA-PEG-RGD, ICG/LAP-PDA-*m*PEG and ILPR NPs with different ICG concentrations (5, 10, 20, 30 and 40 μg/mL). After 24 h incubation, the medium was discarded and the cells were washed with PBS for 3 times, followed by addition of 100 μL fresh DMEM containing 10 μL CCK-8. After incubation of the cells at 37 °C for another 3 h, the absorbance were measured by Thermo Scientific Multiskan MK3 ELISA reader (Thermo Scientific, Hudson, NH) at 450 nm in each well. The standard deviation of 5 wells of each sample was recorded.

In Vitro Cellular Uptake Assay

MDA-MB-231 cells were seeded into 12-well plates at a density of 2×10^5 cells/well at 37 °C and in 5% CO_2 atmosphere overnight, and then the medium was replaced with fresh medium containing ICG/LAP-PDA-*m*PEG and ILPR NPs at different ICG concentrations (5, 10, 20, 30 and 40 μg/mL). After 6 h incubation, the medium was removed carefully and the cells were washed with PBS for 3 times, trypsinized, centrifuged, and counted by Handheld Automated Cell Counter (Millipore, Billerica, MA). The remaining cells were lysed using aquaregia solution (1.0 mL) for 2 days, and then the Si concentration in the cells was quantified by ICP-OES to investigate the cellular uptake of ICG/LAP-PDA-*m*PEG and ILPR.

ROS Production

1,4-diphenyl-2,3-benzofuran (DPBF) was used as a ROS chemical probe to evaluate the $^1O_2$ generation capability of ILPR. Specifically, 2 mL of a DPBF/DMF solution (20 μM) was added to 2 mL of water, LAP, ICG, LAP-PDA-PEG-RGD and ILPR aqueous solution, respectively. The mixture was irradiated by an 808 nm laser (1.2 W/cm^2) for 8 min. Then the absorption intensity of DPBF in different solutions at 417 nm was evaluated by UV-vis spectroscopy. To evaluate the ROS production in cells, MDA-MB-231 cells were seeded into 12-well plates at a density of 2×10^5 cells/well at 37 °C and in 5% CO_2 atmosphere overnight, and then the medium was replaced with

fresh medium containing ILPR or LAP-PDA-PEG-RGD at the same ICG concentrations (10, 40 μg/mL). After 6 h incubation, the medium was removed carefully and the cells were washed with PBS for 3 times. Then the cells were irradiated under an 808 nm laser (1.2 W/cm^2, 5 min). Furthermore, after NIR laser irradiation, 100 μL medium containing DCFH-DA (25 mM) was added in each well for 45 min and then the medium was replaced with PBS.[40] The fluorescence signal in cells was observed by a fluorescence microscopy (CARL Zeiss, Axio Vert. A1, Germany), and the fluorescent intensity was detected using a FACScan Calibur flow cytometer (Becton Dickinson, Mountain View, CA) [41].

PTT and PDT of Cancer Cells in Vitro

To estimate the influence of laser power density on the PTT and PDT therapeutic effect of different materials, MDA-MB-231 cells were incubated with LAP-PDA-PEG-RGD, ICG/LAP-PDA-*m*PEG and ILPR at the same ICG concentration of 40 μg/mL for 6 h, and then irradiated under an 808 nm laser (2.5 cm^2, 5 min) at different power densities (0.8, 1.0, 1.2 W/cm^2). Then, MDA-MB-231 cells were incubated with different materials at different ICG concentrations (10, 20 and 40 μg/mL) for 6 h, and then irradiated under an 808 nm laser (2.5 cm^2, 1.2 W/cm^2, 5 min). The cell viability was evaluated by CCK-8 assay performed by previous protocol and the standard deviation of 5 wells of each sample was recorded.

MDA-MB-231 cells were incubated with LAP-PDA-PEG-RGD, ICG/LAP-PDA-*m*PEG and ILPR at the same ICG concentration of 40 μg/mL for 6 h, and then irradiated under an 808 nm laser (1.2 W/cm^2, 0.25 cm^2, 5 min). Then the cells were stained by PI (8 μM) and Calcein-AM solution (2 μM) for 45 min at room temperature, and rinsed with PBS for 3 times. Finally, the cells were observed via a fluorescence microscopy (CARL Zeiss, Axio Vert. A1, Germany).

Statistical Analysis

One-way ANOVA statistical analysis was performed to evaluate the experimental data. A p value of 0.05 was selected as the significance level, and the data were indicated with (*) for $p < 0.05$, (**) for $p < 0.01$, and (***) for $p < 0.001$, respectively.

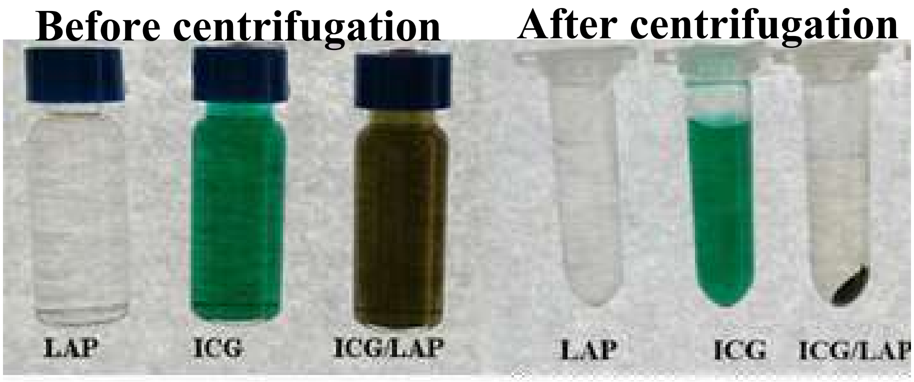

Figure S1. Photographs of ICG, LAP and ICG/LAP before centrifugation (left) or after centrifugation (right).

Table S1. Optimization of ratio for ICG loading (the LAP concentration and ICG concentration were fixed at 2 mg/mL).

Ratio（LAP : ICG）	LE（%）
9 ： 3	54.68
9 ： 2	94.1
9 ： 1	84.46

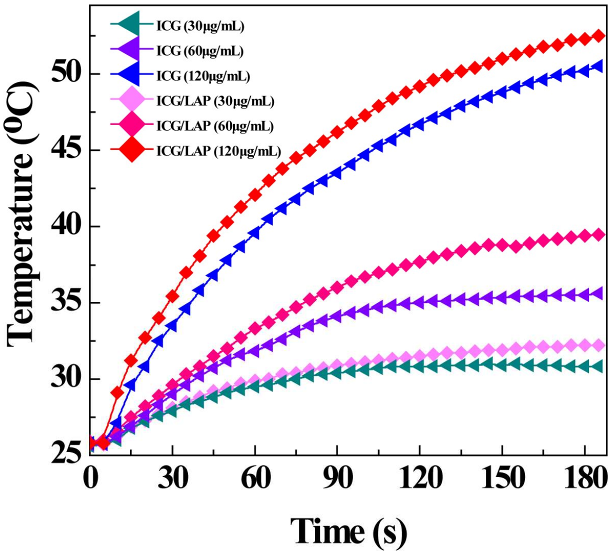

Figure S2. Temperature rising curve of ICG and ICG/LAP at different ICG concentrations (C_{ICG}=120, 60, 30 μg/mL) under irradiation of the 808 nm laser with the power density of 1.2 W/cm^2 for 3 min.

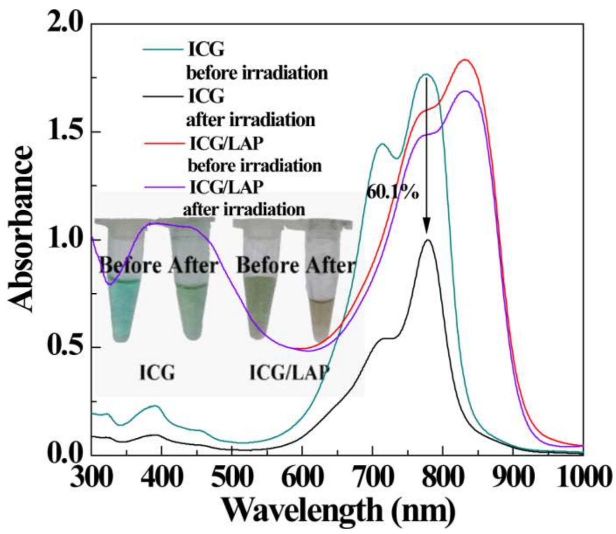

Figure S3. UV-Vis spectra of ICG and ICG/LAP solutions before and after 3 cycle irradiation of 808 nm laser (1.2 W/cm2, 3 min of each cycle). The inset photographs were of free ICG, ICG/LAP and ICG/LAP-PDA solutions before (left) and after (right) irradiation of 808 nm laser for 3 cycles.

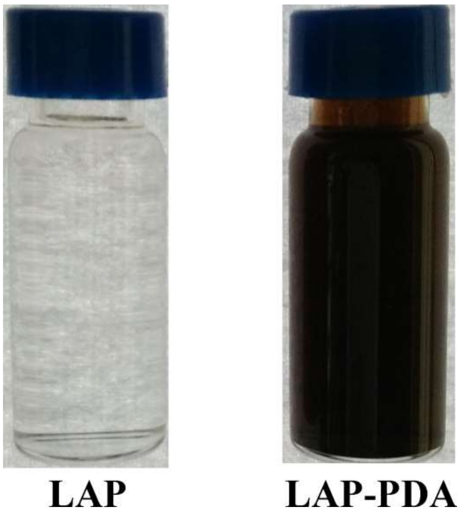

Figure S4. Photographs of LAP (left) and LAP-PDA (right).

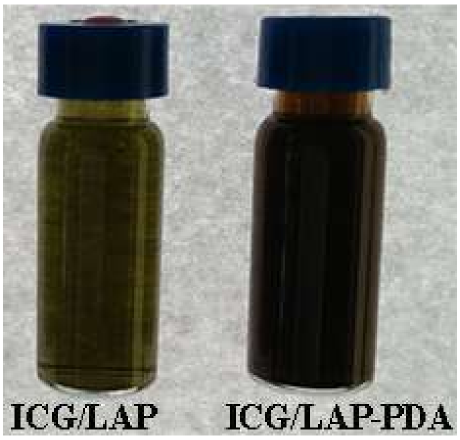

Figure S5. Photographs of ICG/LAP (left) and ICG/LAP-PDA (right).

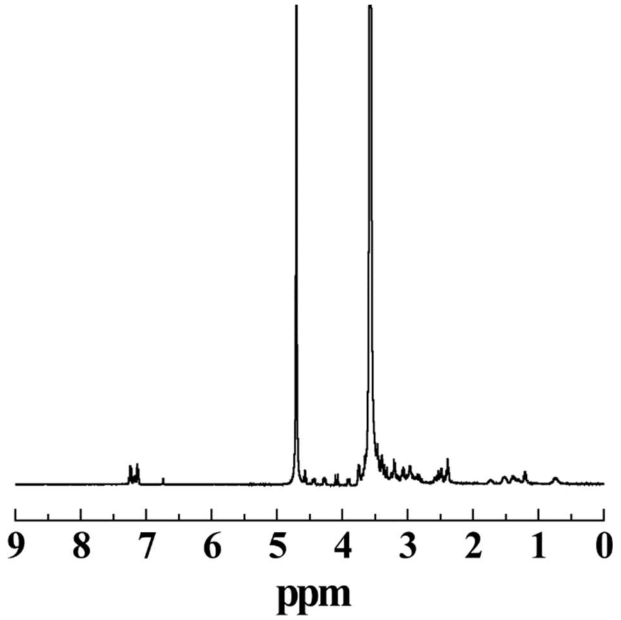

Figure S6. ^{1}H NMR spectrum of the NH_2-PEG-RGD in D_2O.

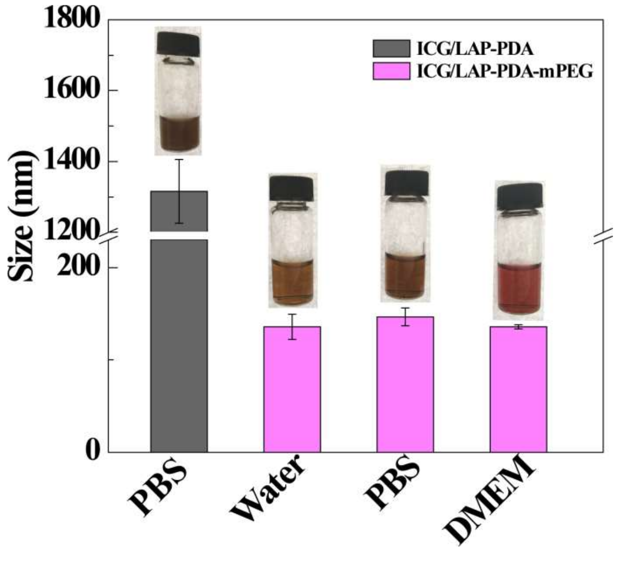

Figure S7. Stability of ICG/LAP-PDA in PBS and ICG/LAP-PDA-*m*PEG in water, PBS and DMEM. The inset photographs were dispersed state of ICG-LAP-PDA in PBS and ICG/LAP-PDA-*m*PEG in water, PBS and DMEM.

LAP-PDA-PEG-RGD were synthesized as a control material without loading ICG and evaluated by DLS (Table S2) and 1H NMR (Fig. S8). After the modification of *m*PEG and PEG-RGD, the hydrodynamic size of NPs slightly increased from 89.2 nm to 104.2 nm and 109.6 nm, indicating that formed nanoparticles still possess favourable size and good distribution for further biomedical application. Meanwhile, the surface potential increased from -32.3 mV of LAP-PDA to -29.5 mV of LAP-PDA-*m*PEG and -27.8 mV of LAP-PDA-PEG-RGD, suggesting the successful modification of PEG chains. The formed LAP-PDA-*m*PEG and LAP-PDA-PEG-RGD were characterized by 1H NMR spectroscopy (Fig. S8). Since the chemical shift of PEG chains at 3.7 ppm overlapped with

that of PDA, an enhanced peak at 3.7 ppm could be observed in the 1H NMR spectrum of LAP-PDA-*m*PEG. After the modification of RGD-PEG, the RGD-associated aromatic proton peaks at 7.3 and 7.4 ppm appeared in the 1H NMR result of LAP-PDA-PEG-RGD, indicating that LAP-PDA-PEG-RGD was synthesized successfully.

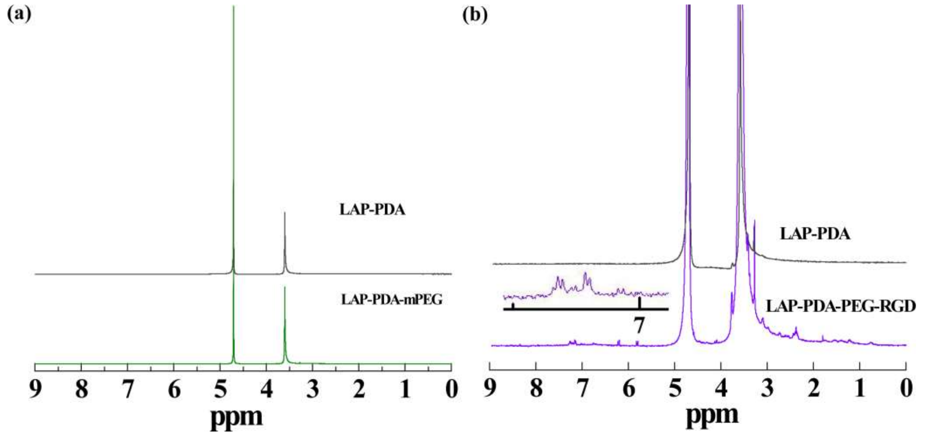

Figure S8. ^{1}H NMR spectra of (a) LAP-PDA, LAP-PDA-*m*PEG and (b) LAP-PDA-PEG-RGD in D_2O.

Table S2. Zeta potential and hydrodynamic size of LAP, LAP-PDA, LAP-PDA-*m*PEG and LAP-PDA-PEG-RGD, respectively.

Materials	Zeta potential (mV)	Hydrodynamic size (nm)	Polydispersity index (PDI)
LAP	-32.5 ± 3.75	62.8 ± 2.74	0.205 ± 0.038
LAP-PDA	-32.3 ± 1.37	89.2 ± 7.64	0.408 ± 0.812
LAP-PDA-*m*PEG	-29.5 ± 0.62	104.2 ± 4.76	0.315 ± 0.043
LAP-PDA-PEG-RGD	-27.8 ± 0.62	109.6 ± 1.15	0.404 ± 0.019
